# Editorial: The Epigenetics in Osteogenic and Chondrogenic Differentiation of Mesenchymal Stem Cells

**DOI:** 10.3389/fcell.2021.784791

**Published:** 2021-10-22

**Authors:** Elena Della Bella, Stefania Pagani, Fernanda Martini, Monica De Mattei

**Affiliations:** ^1^AO Research Institute Davos, Davos Platz, Switzerland; ^2^Complex Structure of Surgical Sciences and Technologies, IRCCS Istituto Ortopedico Rizzoli, Bologna, Italy; ^3^Section of Experimental Medicine, Department Medical Sciences, School of Medicine, University of Ferrara, Ferrara, Italy; ^4^Laboratory for Technologies of Advanced Therapies (LTTA), University of Ferrara, Ferrara, Italy

**Keywords:** epigenetics, osteogenesis, chondrogenesis, mesenchymal stem cells, differentiation

Throughout the lifespan, mesenchymal stem cells (MSCs) provide tissue formation, growth, homeostasis, and renewal. From their discovery (Friedenstein et al., 1968), MSCs have been isolated from different tissues and their properties and abilities have been deeply explored (Pittenger et al., 2019). The intensive investigations have shown the complexity of the mechanisms which control self-renewal, differentiation into different cell phenotypes including bone and cartilage cells and senescence (Zhou et al., 2020). Because of their unique properties, MSCs are attractive tools for cell-based therapy in tissue engineering and regenerative medicine of hard connective tissues. It is now known that MSC differentiation into the osteogenic or chondrogenic lineage is a multistep process, highly regulated by a plethora of specific signaling molecules of the extracellular matrix or produced by neighboring cells, involving multiple extracellular signaling pathways and a complex gene expression regulation (Sagaradze et al., 2020; Chan et al., 2021). A further complexity grade in the regulation of MSCs differentiation is due to several epigenetic mechanisms which have attracted great interest over the last decade. These epigenetic factors or regulators include modifications of histones, adenosine triphosphate (ATP)-dependent chromatin remodeling complexes, DNA methylation, and different classes of non-coding RNAs (ncRNAs) which modulate the expression of a gene by changing the availability of DNA sequences for DNA-binding proteins, inhibiting translation, or cleaving the complementary target messenger RNAs (Iaquinta et al., 2021).

The increasing knowledge in the field of epigenetics stimulated this Research Topic, which aims to collect data showing the impact of epigenetics in osteogenic and chondrogenic differentiation of MSCs. The Topic titled “*The Epigenetics in Osteogenic and Chondrogenic Differentiation of Mesenchymal Stem Cells*” includes comprehensive reviews focused on different critical epigenetic mechanisms involved in MSC differentiation, as well as original research papers. The contribution by Montecino et al. introduces to general mechanisms and molecular complexes which regulate chromatin organization in mammals, thus playing an essential role to differentially control the access to coding genomic sequences. Also, the authors collect recent data demonstrating the role of these epigenetic mechanisms in controlling gene transcription and describe how they contribute to osteogenic differentiation regulating the transcription of *RUNX2* and *SP7* genes, the key osteogenic transcription factors. Another fundamental aspect of the epigenetic regulation of MSC osteogenic differentiation is the regulation of gene expression by several classes of noncoding RNAs. Indeed, it is well-established that both miRNAs and long ncRNAs (lncRNAs) are essential players in gene expression, although their interplay is less known. Lanzillotti et al. provide an exhaustive description of *in vitro* and *in vivo* results concerning the crosstalk between lncRNAs and miRNAs in MSC osteogenic differentiation and their connection with specific components of the osteogenic signaling pathways. Data collected reveal a complex interplay which deserves further investigations. Other regulatory components of RNA stability are RNA binding proteins which act as modulators of gene expression. Among them, Kota et al. have focused on Elavl1 (embryonic lethal-abnormal vision like 1) a highly conserved component of ELAV family proteins with high affinities for U- and AU- rich elements (ARE) containing RNAs. In their study the authors show that Elavl1 is involved in osteogenic differentiation of mouse bone marrow derived mesenchymal stem cells (BMSCs), as Elavl1 knockdown stimulates osteogenic differentiation, in association to the increased stability and expression levels of different mRNAs coding for extracellular matrix components and regulatory enzymes.

The issue of ncRNAs in the modulation of MSC chondrogenic differentiation is addressed in the study by Jiang et al. who investigated the possible function of Super-Enhancer lncRNAs (SE-lncRNAs), a subset of ncRNA transcribed from super-enhancers (Wang et al., 2020). A global picture on potential interconnections among signaling pathways, SE-lncRNAs, and mRNAs associated to the chondrogenic differentiation of BMSCs is shown. The article represents an example of current technologies including microarray and bioinformatic analysis to achieve a bulk of data helpful in identifying possible new chondrogenic key regulators.

The essential role of histone modifications in the regulation of chondrocyte fate, cartilage development and pathologies has been discussed in the contribution by Wan et al. The review mainly focuses on histone-modifying proteins including acetyltransferases (HATs), histone lysine methyltransferases (KMTs), and demethylases (KDMs) and histone deacetylases (HDACs), as well as the effector proteins that recognize modified histones, regulating chondrocyte fate and functions. Emerging evidence shows specific histone changes associated with the expression of key chondrocyte marker genes such as SOX9 and collagen type II and with cartilage pathologies such as osteoarthritis (OA). Interestingly, the work also describes *in vitro* and *in vivo* data concerning the effects of small molecules and drugs able to modify histone signals, introducing to the potential of novel therapeutic approaches for OA and articular cartilage repair or regeneration.

Another intriguing aspect of epigenetics regulation has been reported in the paper by Zhang et al. The authors investigated potential changes of chromatin accessibility under cyclic stretch. Although biophysical stimuli including electromagnetic fields and mechanical forces are known stimulators of MSC differentiation (Schätti et al., 2011; Wang et al., 2016; Martini et al., 2020), their influence on chromatin structure is poorly known. Here it is shown that cyclic stretching favors accessibility of chromatin, particularly of the gene loci associated with MSCs morphogenesis and osteogenesis, increasing knowledge on the molecular mechanisms involved in cellular response to mechanical forces.

Taken together, the papers included in this Research Topic show an emerging fundamental regulatory scenario in which epigenetic mechanisms are strictly interconnected with the more known signals and pathways which drive MSC osteogenic and chondrogenic differentiation. The present collection does not only give an overview of the current understanding of the most known epigenetic mechanisms involved in MSCs differentiation but provides the basis for further studies in this rapidly developing research area. We are confident that future elucidation of epigenetics in skeletal tissues during normal development, as well as in pathological conditions may increase the chance to develop new therapeutic approaches in bone and cartilage diseases.

## Author Contributions

MDM: revision of literature and article drafting. EDB, FM, and SP: revision of literature and final approval. All authors contributed to the article and approved the submitted version.

## Funding

The research of the author affiliated to AO Research Institute Davos (EDB) was supported by the AO Foundation and AO Trauma.

## Conflict of Interest

The authors declare that the research was conducted in the absence of any commercial or financial relationships that could be construed as a potential conflict of interest.

## Publisher's Note

All claims expressed in this article are solely those of the authors and do not necessarily represent those of their affiliated organizations, or those of the publisher, the editors and the reviewers. Any product that may be evaluated in this article, or claim that may be made by its manufacturer, is not guaranteed or endorsed by the publisher.
